# Modification of conducting arylidene copolymers by formation of inclusion complexes: synthesis, characterization, and applications as highly corrosion inhibitors for mild steel

**DOI:** 10.1186/s13065-023-00992-5

**Published:** 2023-07-15

**Authors:** Hemat M. Dardeer, Mohamed Abdel-Hakim, Kamal I. Aly, Marwa M. Sayed

**Affiliations:** 1 Chemistry Department, Faculty of Science, South Valley University, Qena, 83523 Egypt; 2 Chemistry Department, Faculty of Science, Al-Azhar University, Assiut, 71524 Egypt; 3 Polymer Research Laboratory, Chemistry Department, Faculty of Science, Assiut University, Assiut, 71516 Egypt; 4 Chemistry Department, Faculty of Science, The New Valley University, El-Kharja, 72511 Egypt

**Keywords:** Cyclodextrins, Pseudopolyrotaxane, Conductive polymers, Corrosion inhibition

## Abstract

Modifying the metal surface is one solution to the industry’s growing corrosion problem. Thus, via threading approach and insertion of copolymers (CoP5-7) containing polyarylidenes through the internal cavity beta-cyclodextrin β-CD, novel pseudopolyrotaxanes copolymers (PC5-7) are developed, resulting in mild steel corrosion inhibition. Inhibitors of corrosion based on β-CD molecules adsorb strongly to metal surfaces because of their many polar groups, adsorption centers, many linkages of side chains, and benzene rings. The corrosion inhibition efficiencies IE % statistics have been revised via the Tafel polarization method and Spectroscopy based on the electrochemical impedance (EIS), with PC7 achieving the highest 99.93% in 1.0 M H_2_SO_4_; they are mixed-type inhibitors. The chemical composition of the resulting PCs is determined with Fourier transform infrared spectroscopy (FTIR), Scanning electron microscopy (SEM) is utilized to examine the morphological structure of the produced polymers, and X-ray diffraction is employed to identify crystallinity. Encapsulating CoP5-7 with β-CD changes the morphological structures and increases the generated PCs' crystallinity. The thermal stability of PCs is studied, indicating the presence of these CoPs within the β-CD cavities enhances their thermal stability. This research will be a stepping stone for developing high-efficiency anti-corrosion coatings and various industrial applications.

## Introduction

The delocalization of π bonds in conducting polymers (CPs) relies on the disorder of their chains and generates charge carriers to transfer their nature to be a metal [1]. They have broad and significant applications in different fields and problems, including the corrosion problem. This trouble is considered serious and can extremely produce loss and environmental hazards. CPs can reduce corrosion by keeping the metal passive through different mechanisms [2, 3]. Their use as inhibitors has some limitations because of their poor adhesion, sponginess, and irreversibility of charge consumption. The direction toward modification of CPs is continued and in progress by different techniques from nano to composites [4, 5].

Overcoming the corrosion of several kinds of steel is the fundamental issue facing the industrial sectors. By adding corrosion inhibitors, steel is effectively protected against corrosion during acid pickling to remove mill scale from metallic surfaces. It is possible to use several conventional corrosion materials as inhibitors. However, using this kind of inhibitor raises concerns about environmental safety. The majority of organic corrosion inhibitors are made via numerous phases of reactions with traditional heating, hazardous and expensive chemicals, solvents, and catalysts. The classic organic corrosion inhibitors also have fewer adsorption sites in their molecular architectures, which results in inadequate metal surface coverage. Additionally, they only partially dissolve in polar electrolytes. Therefore, only at increasing concentrations do these chemicals start to work [6].

Many efforts have recently been undertaken to discover novel natural and biodegradable compounds that can be corrosion inhibitors but don’t hurt people or the environment. The fact that polysaccharides have active sites interacting with metal ions makes them potential green and natural inhibitors [7].

Macrocyclic materials are large planar systems, and their derivatives, such as crown ethers, phthalocyanines, porphyrin rings, calixarenes, and cyclodextrins, are among the substances most commonly used as effective corrosion inhibitors [8].

One of these strategies is the use of cyclodextrins (CDs) to modify the solubility or form an insulated molecular wire through polyrotaxane formation (PRs) by preventing their oxidation by losing their delocalization [9–11]. CDs are a type of cyclic oligosaccharide made up of 6 to 8 glucopyranose units, designated by the letters α*‚ β*- and γ CD. They have a unique structure with an internal surface resembling a cavity with hydrophobic characteristics. They could contain various organic and polymeric compounds or complexes that can form inclusions with organic molecules in an aqueous medium [12]. CD inner diameters are − 0.47, − 0.60, and − 0.75 nm, with a cavity depth of 0.79 nm. CDs are essential in the improvement of supramolecular chemistry and have important uses in material science, such as rigid polymers [13, 14], therapeutic science [15, 16], and cosmetics [17]. The dominant driving forces for creating supramolecular structures are the hydrophobic interaction and the hydrogen bonding in the crystal structure and the hydrous solutions [18, 19]. Cyclodextrin, the most common kind, comprises seven units of glucopyranose and two types of hydroxyl groups, secondary and primary groups, resulting in a macromolecule with twenty-one hydroxyl groups in total [20].

Supramolecular (host–guest) systems based on polymer β-cyclodextrin (CD) are used to develop new materials with desirable properties for green corrosion inhibitors. These materials have a high inhibitory efficiency while being durable, inexpensive, eco-friendly, and user-friendly. In contrast to other interlocked compounds made up of polymer chains and macrocyclic molecules, polyrotaxanes (PRs) have intramolecular mobility characterized by rotation and sliding of the rings along the chains [21]. Supramolecular complexes based on CD derivatives like octadecyl amine [22, 23], organic compounds [24–26], and also CD modified with graphene produce effective corrosion Inhibitors [27]. There are a few considerations about conducting polymer and Cyclodextrin using CDs as a template molecule, dopant, or conformation converter [28–31].

The main goal of this investigation is to create and innovate new types of corrosion inhibitors that rely on β-CD macromolecules by modifying the corrosion inhibition of conducting copolymers that have already been produced, adding β-CD rings, and then analyzing the results to see how this inclusion affects the characteristics of the conducting copolymers and the corrosion efficiency.

## Experimental

### Materials

β-cyclodextrin (β-CD) (Merck company, Germany), dimethylformamide (DMF) (Aldrich, Milwaukee, Wisconsin, USA) without additional purification, cyclopentanone, cyclohexanone, and cycloheptanone (Merck) were employed without crystallization, terephthaldehyde (Alfa Aesar) was utilized without further purification, sulfuric acid (H_2_SO_4_) was used as an acid medium (purchased from PROLABO), all chemical compounds were used as obtained.

### Synthesis of copolymers (CoPs5-7)

**Scheme 1:**
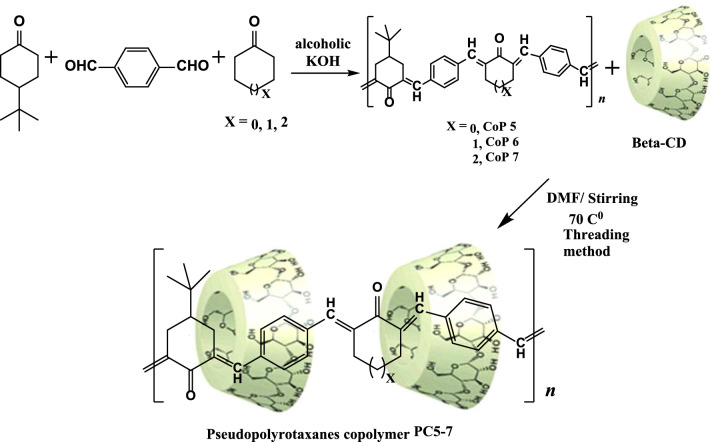
Synthetic method for preparing (CoP 5–7) and pseudopolyrotaxanes (PC5-7)

The copolymerization of terephthaldehyde with various cycloalkanones (cyclopentanone, cyclohexanone, and cycloheptanone) was previously reported [32–37]. The synthesis of copolymers is shown in (Scheme 1) as a synthetic approach (CoP5-7).

### Synthesis of pseudopolyrotaxane copolymer (PC5)

The copolymer (CoP 5) (1.5 g) was dissolved in 15 mL DMF before adding to the β -CD solution (5 g, 4.40 mmol in 30 ml DMF) at 70 °C; the reaction mixture was agitated for 22 h. The precipitate was filtered off, washed with distilled water, and dried as yellow PC5 (86% product, m.p. over 300 °C).

### Synthesis of pseudopolyrotaxane copolymer (PC6)

The copolymer (CoP 6) (1.0 g) was dissolved in 10 mL DMF before adding to the β-CD solution (3 g, 2.64 mmol in 20 ml DMF) at 70 °C; the reaction mixture was agitated for 18 h. The resulting precipitate was filtered out, washed with distilled water, and dried to yield the (PC6) deep yellow residue (77% yield, m.p. over 300 °C).

### Synthesis of pseudopolyrotaxane copolymer (PC7)

The copolymer (CoP 7) was dissolved in 30 mL DMF and added to the β-CD solution (3.5 g, 3.08 mmol in 20 ml DMF) at 70 °C; the reaction mixture was stirred for 24 h. Until the formation of yellow precipitate. The (PC7) was filtered out and dried in a 75% yield, m.p. over 300 °C.

### Preparation of the corrosive solution and test specimens:

The corroding media in electrochemical experiments was Analar grade H_2_SO_4_ and was applied as an aerated solution. The working electrode was mild steel; all analyses were performed and accomplished in Cairo at the Tabbin Institute for Metallurgical Studies. Mild steel components are 0.022% F, 0.011% Mo, 0.011% Sn, 0.17% C, 0.71% Mn, 0.022% Si,0.045% Cr, 0.010% P, 0.0017% Al, 0.072% Ni, 0.182% Cu, and 98.74% Fe. The electrode was polished with emery paper ranging from 1000 to 1400 grade, then cleaned with distilled water before degreased with acetone. After about 5 min, rinsed again with distilled water and dried using filter paper [38].

### Preparation of corrosive and inhibitor solutions

All of the corrosion inhibitors in the study were made by dissolving copolymer and polyrotaxane in DMF (200 ppm). The electrochemical tests were performed by pickling a mild steel electrode for 15 min in the produced corrosion inhibitors solution, then drying and immersed in 1.0 M H_2_SO_4_ (25 mL) [39].

### Measurements

Before analysis, the samples were carefully dried under a vacuum to eliminate residual solvent molecules. The chemical structures of the synthesized pseudopolyrotaxane copolymers were proved using Fourier transform infrared (FT-IR) spectroscopy. An infrared spectrometer (Jasco Model 4100—Japan) with a wavenumber range of 4000–500 cm^−1^ was used to analyze the functional groups in the generated pseudopolyrotaxane copolymers at room temperature. The morphological structures of the generated pseudopolyrotaxane copolymers were revealed using SEM imaging with the Jeol JSM-5400 LV instrument (SEM). A powder diffractometer (Brucker D8 Advance, Germany) with Cu K radiation source, λ = 1.5406 and 2θ in the range (5–80°) was used to measure X-ray diffraction bands at indoor temperature to study the specimen's phase and crystalline structure. The Thermal Analyzer TA Q-600 measures thermogravimetric analysis (TGA) at a 10 °C/min rate in an N_2_ environment.

### Electrochemical measurements

All electrochemical experiments, open circuit potential (OC), linear and Tafel polarization (LP, TP) were carried out by electrochemical instrument (EG & G potentiostat/galvanostat model 273A) using a 352/252 corrosion measurement system. The steady-state potential E_s.s_ (open circuit potential technique) were immediately evaluated after pickling the working electrode(mild steel) within the prepared solutions, followed by potentiodynamic polarization parameters (LP, TP). electrochemical impedance spectroscopy (EIS) was measured through (Corrtest CS350). The linear polarization (LP) and the Tafel polarization (TP) were determined in the range (of + 20 to − 20 mV) and (− 250 to + 250 mV) using scan rates of 0.166 and 0.4 mV/S, respectively. The rate of corrosion (CR) and the corrosion inhibition efficiency percentage (IE %) were detected using Eqs. 1 and 2 [40].

1 $${\text{CR}} = 0.13\left[ {\frac{{\left( {{\text{Eq}}.{\text{wt}}} \right)\left( {I_{{{\text{corr}}}} } \right)}}{{\left( {d \times A} \right)}}} \right]$$

where C.R (mpy): corrosion rate (millimeters per year), Icorr: corrosion current density (μA/cm^2^), (Eq. Wt): equivalent weight of the metal (g/eq), A: surface area (cm^2^), d: density (g/cm^3^), and 0.13: metric and time conversion factor.

2 $$IE{\text{\% }} = \left[ {\frac{{\left( {I_{without\,inhib. - } I_{with\,inhib. } } \right)}}{{I_{without\,inhib. } }}} \right] \times 100$$

where (CR uninh, CR inh): corrosion rates without or with inhibitors, respectively.

## Results and discussion

For organic compounds to work as corrosion inhibitors, they need oxygen atoms that can easily stick to the surface of the metal. This is done by forming coordination bonds between the metal and the organic compounds’ unshared electron pair. The prepared supramolecular copolymers' chemical structure has many carboxyl and hydroxyl groups. As a result, the supramolecular copolymers’ active groups can attach to the metal’s surface and prevent corrosion. In addition to pseudopolyrotaxanes, β-CD copolymers can prevent metal from corroding at both high and low temperatures. This is because they are soluble and have π-bonds. Also, these compounds are made in ways that are good for the environment and don’t cost much. So, these compounds are considered the next generation of chemicals that stop corrosion [41, 42].

### FT-IR spectroscopy

**Fig. 1:**
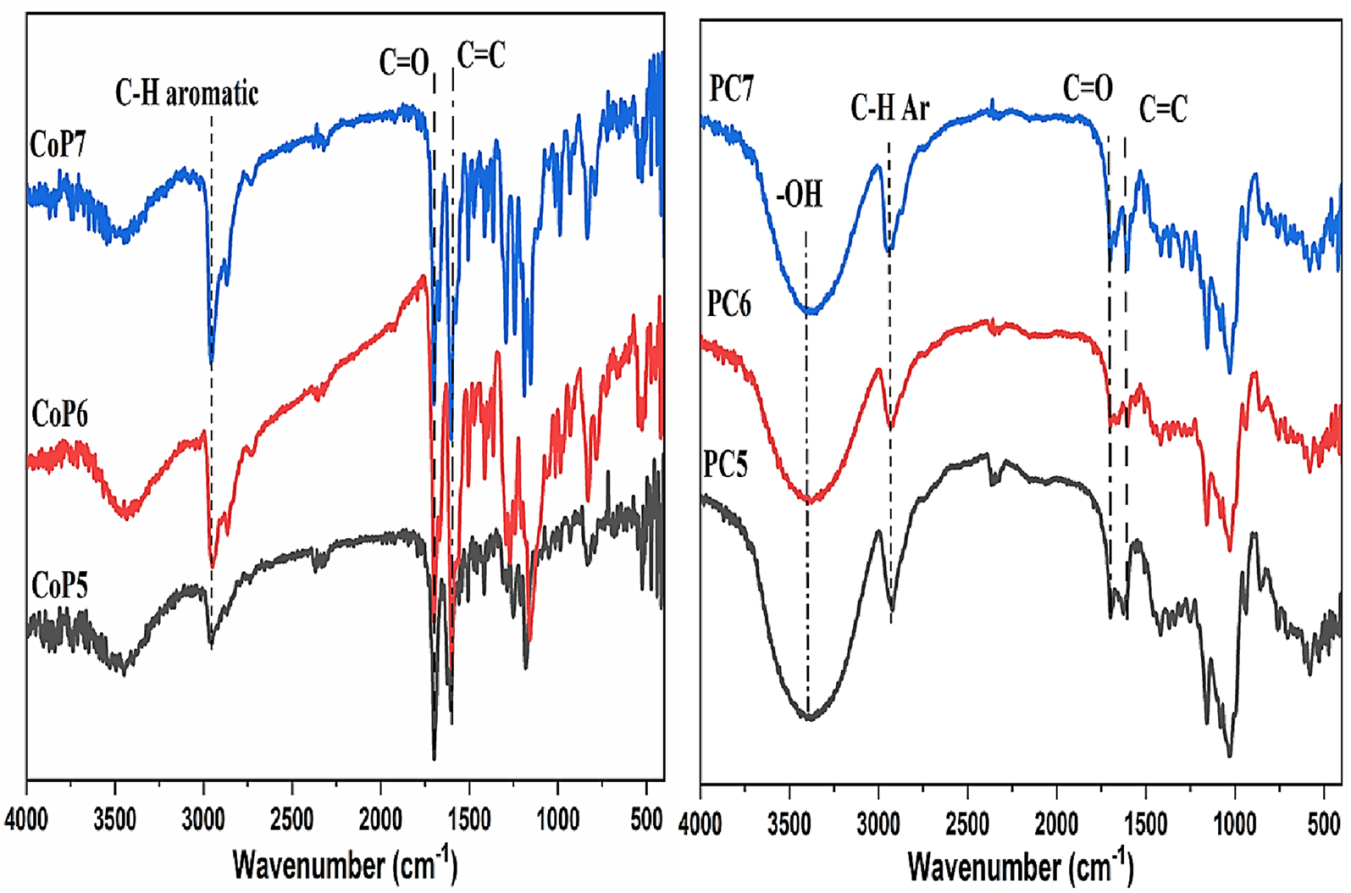
FTIR spectra for (CoP5-7) (Left), (PC5-7) (Right)

The threading approach was utilized to synthesize pseudopolyrotaxane copolymers (PCs5-7). β–CD rings include the (OH) groups that raise coordination bonds with metal surfaces and decrease corrosion development accordingly. The unique cone-shaped structure of β–CD makes it acceptable to construct inclusion complexes by encapsulating the arylidene copolymers (CoP5-7) into its cavity to yield the different polyrotaxanes by the complex inclusion method. This behavior achieves and stabilizes by creating non-covalent bonds like Vander Waals forces, hydrogen bonding, and hydrophobic-hydrophobic interactions between the hydrophobic cavity and the hydrophobic moieties of conducting arylidene Copolymers chains (aliphatic cyclic chains and aromatic rings). The chemical functional group's features and vibration were examined using IR spectroscopy. (Fig. 1) compares the FTIR spectra of the prepared (CoP5-7) and the new pseudopolyrotaxane copolymers (PCs5-7). The FTIR spectrum of (CoP5) affords peaks at 2891, 3010, 1726 and 1664 ${\mathrm{cm}}^{-1}$, represent CH aliphatic, CH aromatic, C=O group, and C=C, respectively. Meanwhile, the FTIR spectrum of (PC5) shows bands at 2885, 3007, 1734, and 1669 cm^−1^ due to CH aliphatic, CH aromatic, C=O group, and C=C, respectively. The broadband in the (PC5) spectrum at $\sim$ 3393 cm^−1^ attributes to stretching vibrations of (OH) groups intermolecular hydrogen bonding in β–CD rings also, between the carbonyl group in (PC5). The FTIR spectrum of (PC5) indicates some spectral variations like energy shifting towards the lower wavenumber for (CH aliphatic and CH aromatic) and their intensity increase. In contrast, the bands for (C=O and C=C) were shifted toward high frequencies with less intensity. In the FT-IR spectrum (CoP6), the peaks for its active groups appear at 2843, 3012, 1743, and 1674 cm^−1^ due to CH aliphatic, CH aromatic, C=O group, and C=C, respectively. In addition, the bands for (PC6) appeared at 2854, 3018, 1736, and 1671, respectively, with increasing and decreasing intensity; this result is due to the overlapping between carbonyl groups with OH groups. Also, the hydrogen bond interaction between the carbonyl groups of the guest molecule and the (OH) groups of the β-CD ring causes the displacement of the C=C and carbonyl groups to higher wavenumbers and boosts their intensity [37, 43–46]. Due to the inclusion of (CoP6) inside the hydrophobic cavity of β-CD, the distinctive absorption band due to (OH) groups of (PC6) became sharper than pure β-CD [47]. Interactions between the ether linkage inside the cavity and the benzene rings are also hydrophobic-hydrophobic (CoP6) [48].

FT-IR spectra of the obtained (PC7) are displayed in (Fig. 1), which shows that the peak of the aliphatic CH symmetric stretching and CH aromatic of the (CoP7) were present at 2843, 3006 cm^−1^ and were shifted to higher frequencies. After complex inclusion, the peaks of [C=O] were moved to lower frequencies due to hydrogen bonding between OH groups of β-CD and CO groups of (CoP7). The variations of (CoP5-7) frequencies and (PC5-7) are presented in (Table 1), which describes the frequency changes of (CoP5-7) and (PC5-7) when the inclusion complex is created (PC5-7). These results could mean that (CoP5-7) and β-CD join together to make a complexation. The maximum value is raised by incorporating (CoP5-7) chains into the cyclodextrin rings' electron-rich cavity [37, 43]. Instead, hydrogen bonding between neighboring β-CD rings and Vander Waals forces between the OH groups of β-CDs and CO groups of copolymers are responsible for the attenuation of peaks.

**Table 1:** The difference in the intensity of pure *β*–Cyclodextrin (*β*–CD), Copolymers (CoP5-7), and pseudopolyrotaxane copolymer (PC5-7)

IR-vibration	Wavenumber (cm^−1^)		Δδ	IR-vibration	Wavenumber (cm^−1^)		Δδ
	*β*-CD	PC5			CoP 5	PC5	
ν[OH] symmetric	3389.28	3393.13	+ 3.85	ν[CH] aliphatic	2891.73	2885.95	− 5.78
ν[CH]aliphatic	2925.48	2920.66	− 4.82	ν[CH] aromatic	3010.33	3007.44	− 2.89
ν[C-O-C]	1158.55	1155.15	− 3.4	ν[C-C]	1664.37	1669.61	+ 5.24
ν[O-H] β	1027.87	1023.05	− 4.82	ν[C-O]	1726.94	1734.47	+ 7.53

IR-vibration	Wavenumber (cm^−1^)		Δδ	IR-vibration	Wavenumber (cm^−1^)		Δδ
	*β*-CD	PC6			CoP 6	PC6	
ν[OH] symmetric	3389.28	3391.20	+ 1.92	ν[CH] aliphatic	2843.52	2854.13	+ 10.61
ν[CH]aliphatic	2925.48	2916.80	− 8.68	ν[CH] aromatic	3012.26	3018.05	+ 5.79
ν[C–O-C]	1158.55	1165.75	+ 7.2	ν[C-C]	1674.87	1671.73	− 3.14
ν[O–H] β	1027.87	1024.65	− 3.22	ν[C-O]	1743.33	1736.58	− 6.75

IR-vibration	Wavenumber (cm^−1^)		Δδ	IR-vibration	Wavenumber (cm^−1^)		Δδ
	*β*-CD	PC7			*β*-CD	PC7	
ν[OH] symmetric	3389.28	3393.13	+ 3.85	ν[CH] aliphatic	2831.95	2843.52	+ 11.57
ν[CH]aliphatic	2925.48	2920.66	− 4.82	ν[CH] aromatic	3015.15	3006.84	− 8.31
ν[C-O-C]	1158.55	1155.15	− 3.4	ν[C-C]	1680.65	1689.33	+ 8.68
ν[O-H] β	1027.87	1023.05	− 4.82	ν[C-O]	1739.47	1730.79	− 8.68

### X-RD analysis

**Fig. 2:**
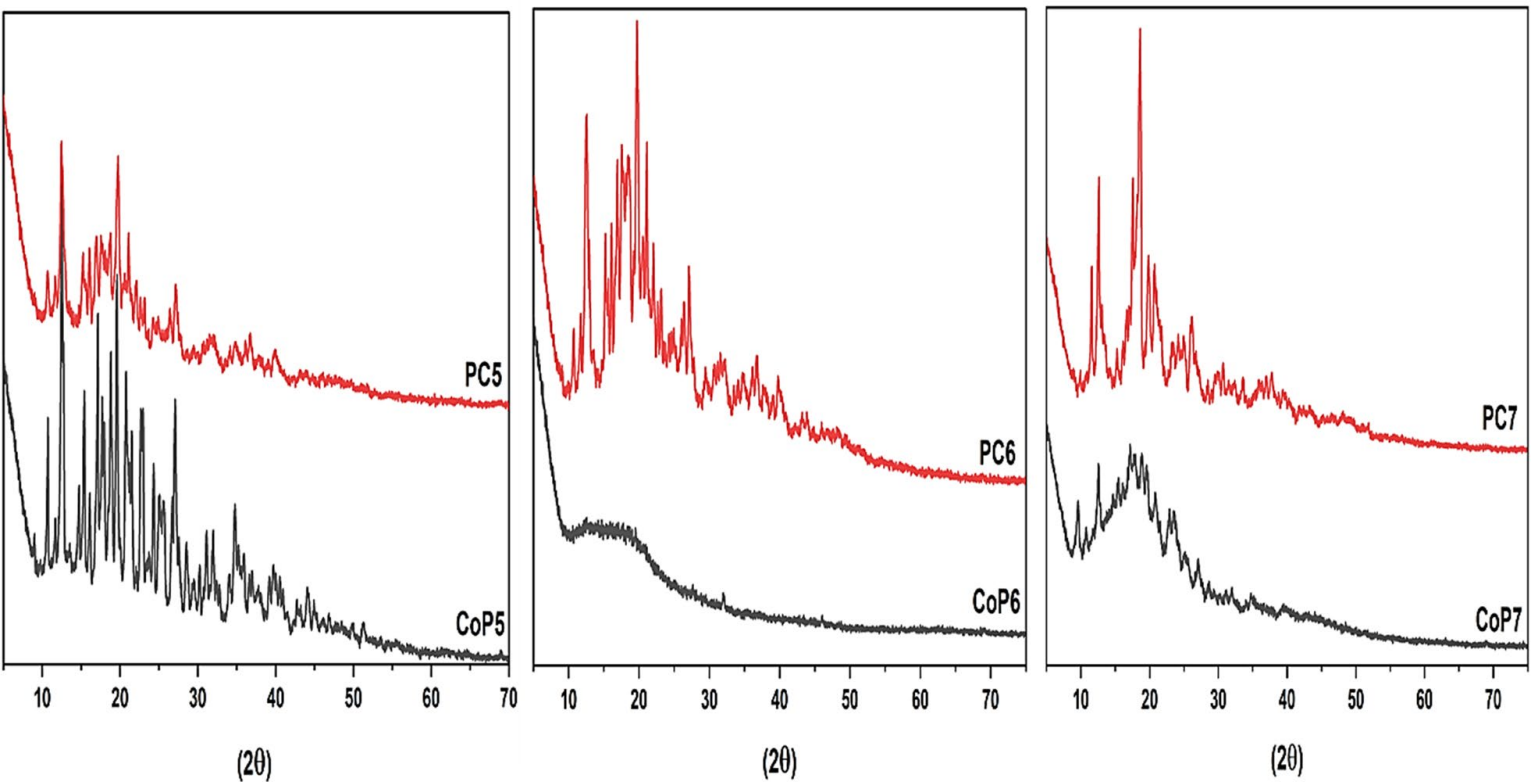
X-RD spectra of (CoP5-7) and (PC5-7)

After making inclusion complexes by putting copolymer chains into the hydrophobic cavity of cyclodextrins, the inclusion shifts the diffraction peak to smaller angles and changes the d-spacing. X-ray diffraction is an excellent and vital way to figure out how the crystal structures of copolymers (CoP5-7) and inclusion complexes are different (PCs). The X-ray patterns of the copolymers (CoP5-7) compared to (PC5-7) are shown in (Fig. 2). Copolymers (CoP5-7) have crystallinity values of 82.4, 31.8, and 59.8%, respectively. Pseudopolyrotaxane copolymers (PC5-7) have crystallinity values of 75.8, 58.8, and 70%, respectively. Planarity and structural effect are responsible for the significant rise in (CoP6,7) crystallinity and the slight decrease in (CoP5) crystallinity. The PC5 crystallinity diminishes because the monomer molecules' planarity reduces as the cyclic ketone size grows. [39]. Due to planarity, copolymer layers stack, and crystallinity rises; hence the structure of CoP5 is more crystalline than its PC5 counterpart, which decreases crystallinity and prevents stacking in contrast to other samples [49]. CoP6,7 inclusion complexes are channel types with an extended column structure, whereas CoP5 is a cage-type [50, 51].

We can extrapolate that complex formation, phase structure, and crystallite size (PC5-7) improve (CoP5-7).

### SEM analysis

**Fig. 3:**
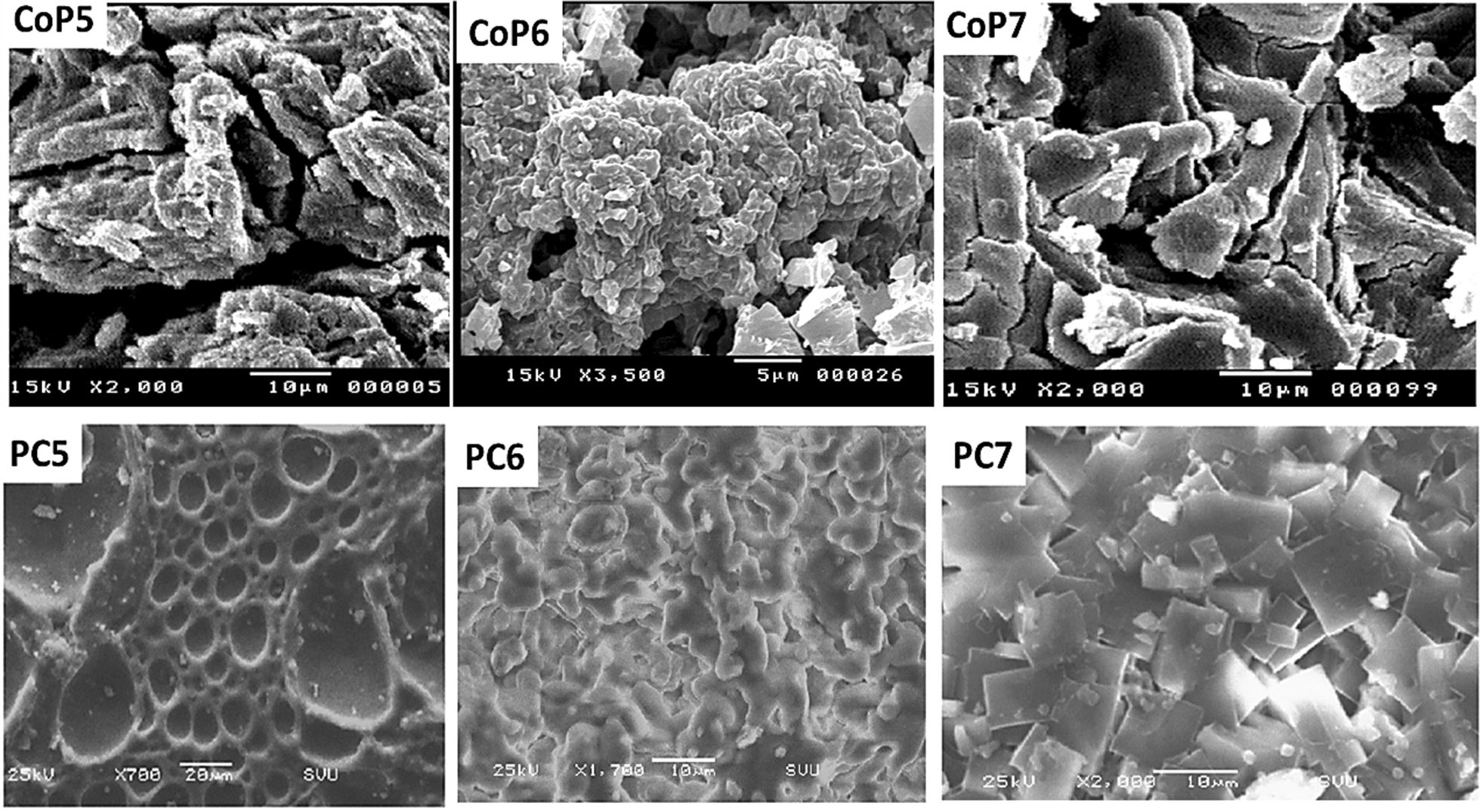
SEM images of (CoP5-7) and (PC5-7)

The structures and morphology of (CoP5-7) and (PC5-7) were observed by SEM analysis, as revealed in (Fig. 3). The copolymer (CoP5-7) possessed an amorphous and grainy structure from the previous work [39]. In contrast, the structures of (PC5-7) were changed. The morphology structure of (PC5) has a regular shape, like holes. In addition, SEM scans of the (PC6) matrix revealed a consistent, smooth, and pore-free surface. This architecture shows the integration of β-CD into the chain of copolymers; the regularity results from creating an inclusion complex. Furthermore, the surface morphology of (PC7) was presented as characteristic slides.

### Thermogravimetric analysis:

**Fig. 4:**
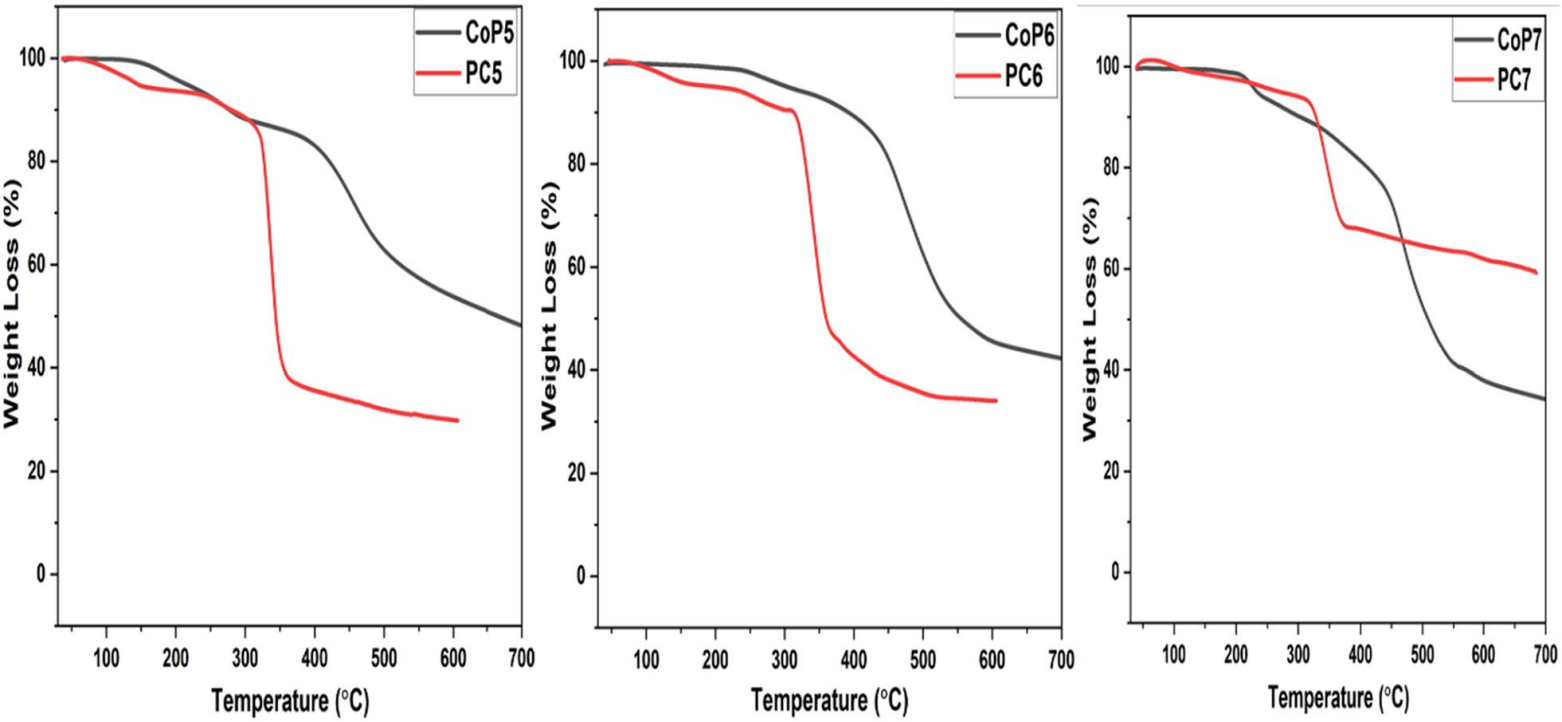
TGA curves of (CoP5-7) and (PC5-7)

The thermogram of the TGA. PC5-7 and CoP5-7 displayed a three-step weight decrease (Fig. 4). The initial loss of weight was attributable to solvent and water evaporation. In contrast, the second and third weight losses were caused by CD degradation and aromatic residue breakdown, respectively, resulting in char formation (Table 2). The inclusion of CD complexes decreases the degradation temperature and increases the decomposition rate. Furthermore, the final T_d_ increased considerably during composite production with CD in PC7; all CoP5-7 exhibits greater degradation temperatures than their respective PC5-7 and a high char yield at 600 °C, except for PC7; it is evident that the structure of CoP5-7 and CD has a discernible effect on their thermal stability. However, PC7 was expected to have the maximum char yield among the PRCP; the existence of a cycloheptanone moiety next to the aromatic carbons improved the polarity of the monomer molecule. According to prior quantum calculations, CoP7 has a greater dipole moment of 2.6 in the trans conformer than CoP5 and CoP6, which have dipole moments of 1.4 and 0.1, respectively, demonstrating its greater polarity and more bonding between CD than the other CoP5,6 [39]. Due to the hydrogen bond between CD and the guest molecule, the degradation temperature generally rises as polarity increases, hence thermal stability [52]. These data provide additional proof of the development of PC5–7.

**Table 2:** Temperature (°C) for various decomposition levels in N_2_ at a heating rate of 10 °C/min

Polymer	10% wt. Loss	20% wt. Loss	30% wt. Loss	40% wt. Loss	50% wt. Loss	Char yield (%) at 600 °C
CoP5	277	421	464	523	665	54
PC5	279	327	333	338	344	30
CoP6	392	453	479	508	554	46
PC6	313	330	339	348	360	34
CoP7	303	411	458	480	509	38
PC7	328	347	367	669	> 685	62

### Electrochemical examinations

#### Open circuit potential estimations

**Fig. 5:**
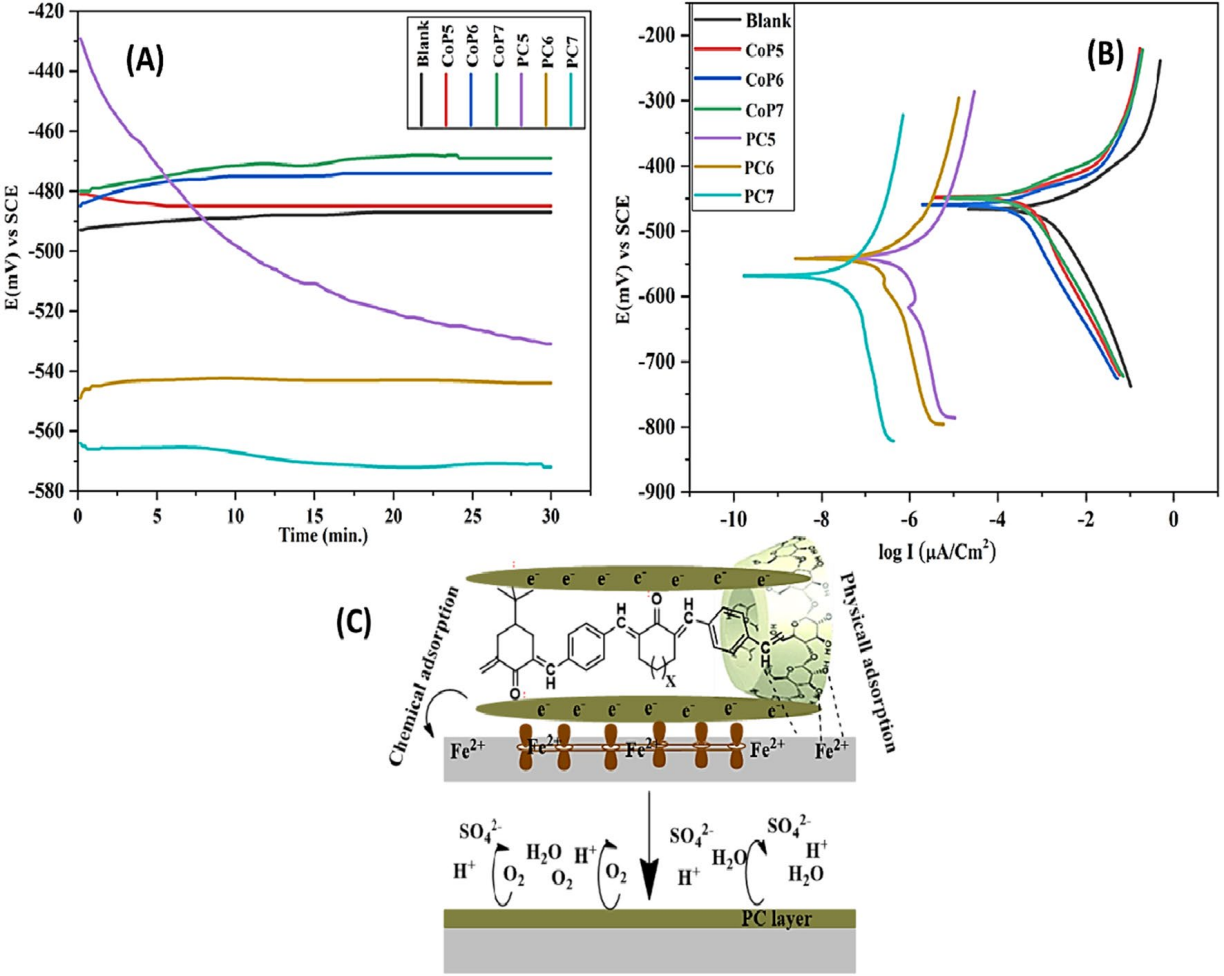
**A** Potential (mV) against Time (min.). **B** Potentiodynamic polarization of mild steel exposed to CoP5-7 and PC5-7 inhibitors with the blank solution. **C** Mechanism of inhibition of PC on mild steel

The open-circuit technique is the relationship between time (t) and potential (E) in the absence of counter electrodes to stabilize the potential of electrochemical cells. (Fig. 5) describes all inhibitors or blanked solutions' steady-state potential (Es.s). It leads to incorporating an oxide film of mild steel electrodes surface. A blank solution’s steady-state potential (Es.s) altered significantly positively relative to its immersion potential (E_im_.). These changes are attributable to the modified copolymers (PC5–7) adsorbed on active surface sites.

#### Polarization measurements

Previous research has shown that steric parameters, electron density, active groups, metal type, and organic-metal connections affect the mechanism by which organic molecules bind to a metallic surface. Physical contact is the initial step in this phenomenon [53, 54]. Donor–acceptor interactions between unoccupied electron pairs of heteroatoms or electrons of multiple bonds and between vacant d orbitals of metals lead to the chemical adsorption of organic compounds. As shown in (Fig. 5), the inhibitory mechanism of PC5-7 on the mild steel surface primarily involves the -CO- groups and conjugated electrons of PCs molecules, which serve as anodic adsorption sites. Anodic adsorption centers block cathodic reactions that result in the evolution of hydrogen. The hydroxyl -OH groups of β-CD served as the physical adsorption centers because they donate more electrons. Electrostatic repulsion between the -OH groups on the metal surface and the corrosion inhibitors caused a reaction. The rate of anodic disintegration decreased dramatically [55–57].

Tafel polarisation (TP) provides a visual representation of the method by which corrosion inhibition occurs as a relation between Log I (μA/ cm^2^) and potential E. (mv). The potentiodynamic polarization of studied corrosion inhibitors (PC5-7) in 1.0 M H_2_SO_4_ compared with reviewed (CoP5-7) [39] is displayed in (Fig. 5). The parameters of electrochemical polarization measurements are shown in (Table 3), corrosion current density (I_corr_), rate of corrosion (CR), and effectiveness in preventing corrosion (IE%). The inhibition efficiencies (IE %) of reviewed (CoP5-7) depend on the chemical composition and the chain length that they are increased by increasing the molecular weight from CoP5 to CoP7 (85.2–87.8%), respectively [39]. Introducing β-Cyclodextrin to the chain of (CoP5-7) by the threading method that is mentioned above yields excellent corrosion inhibitors (PC5-7) with IE % (99.88–99.93). The remarkable efficacy of inhibition of modified (PC5-7) was enhanced by the addition of β-cyclodextrin to the chain of (CoP5-7), which is characterized by a large bulky polymer, hydrophobic cavities, mild steel has a thin layer of Fe_2_O_3_ on its surface, and modified inhibitors hold to it very well. Indicating the increased adsorption of particular PCs on mild steel, the degree of surface coverage measure (θ = IE%/100) was the most helpful in explaining the enhanced corrosion prevention [58]. The surface coverage increased from PC5 to 7 due to the steady rise in polarity of CoP5-7 that altered the interaction between them and β-CD, resulting in molecules with high insulation and complexation that is responsible for their increasing inhibiting PCs from 5 to 7, which covered a vast metal surface area [59]. The distinction between the corrosion potential of the blank solution and the solution containing inhibitors less than 85 mv leads to the classification of the inhibitors as mixed inhibitors [60].

**Table 3:** Potential (mV)- time (min.), polarization parameters of linear polarization, and Tafel plots for CoP5-7 and PC5-7 with the blank solution

Inhibitors name	E_im_(mV)	E_s.s_(mV)	I(µA/Cm^2^)	CR	IE%	(θ)
Blank solution	− 493	− 487	2800	2582	–	–
CoP5	− 481	− 485	400	369	85.2	0.852
CoP6	− 485	− 474	330	304	87.8	0.878
CoP7	− 480	− 469	290	267	89.3	0.893
PC5	− 429	− 531	3.3	3	99.88	0.998
PC6	− 549	− 544	2.9	3	99.90	0.999
PC7	− 564	− 572	1.9	2	99.93	0.999

**Fig. 6:**
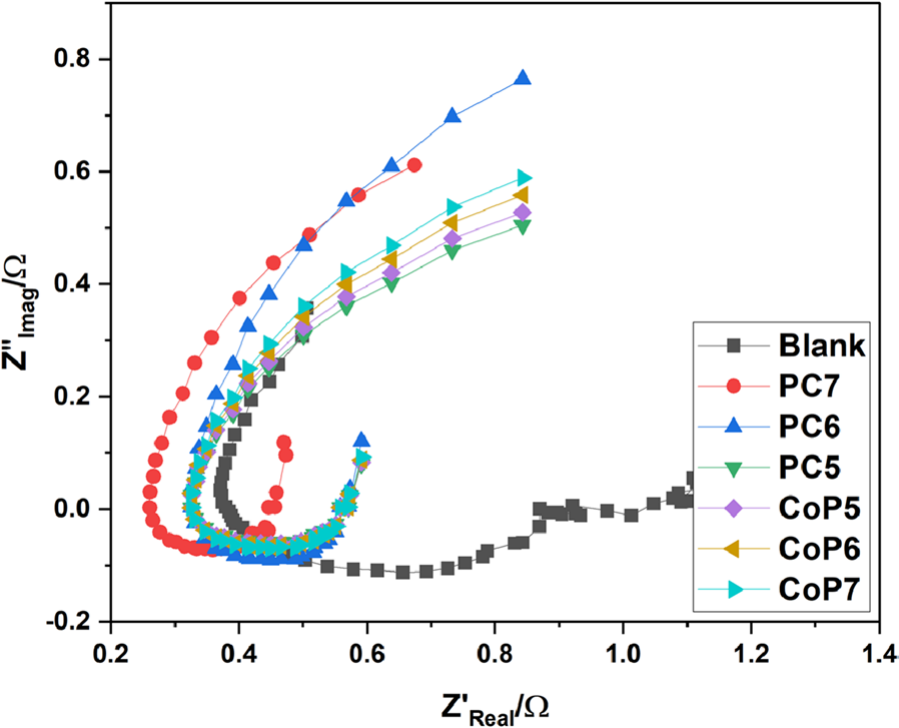
Nyquist plot for Mild steel corrosion in different samples of CoP5-7 and PC5-7 at 303. K

**Fig. 7:**
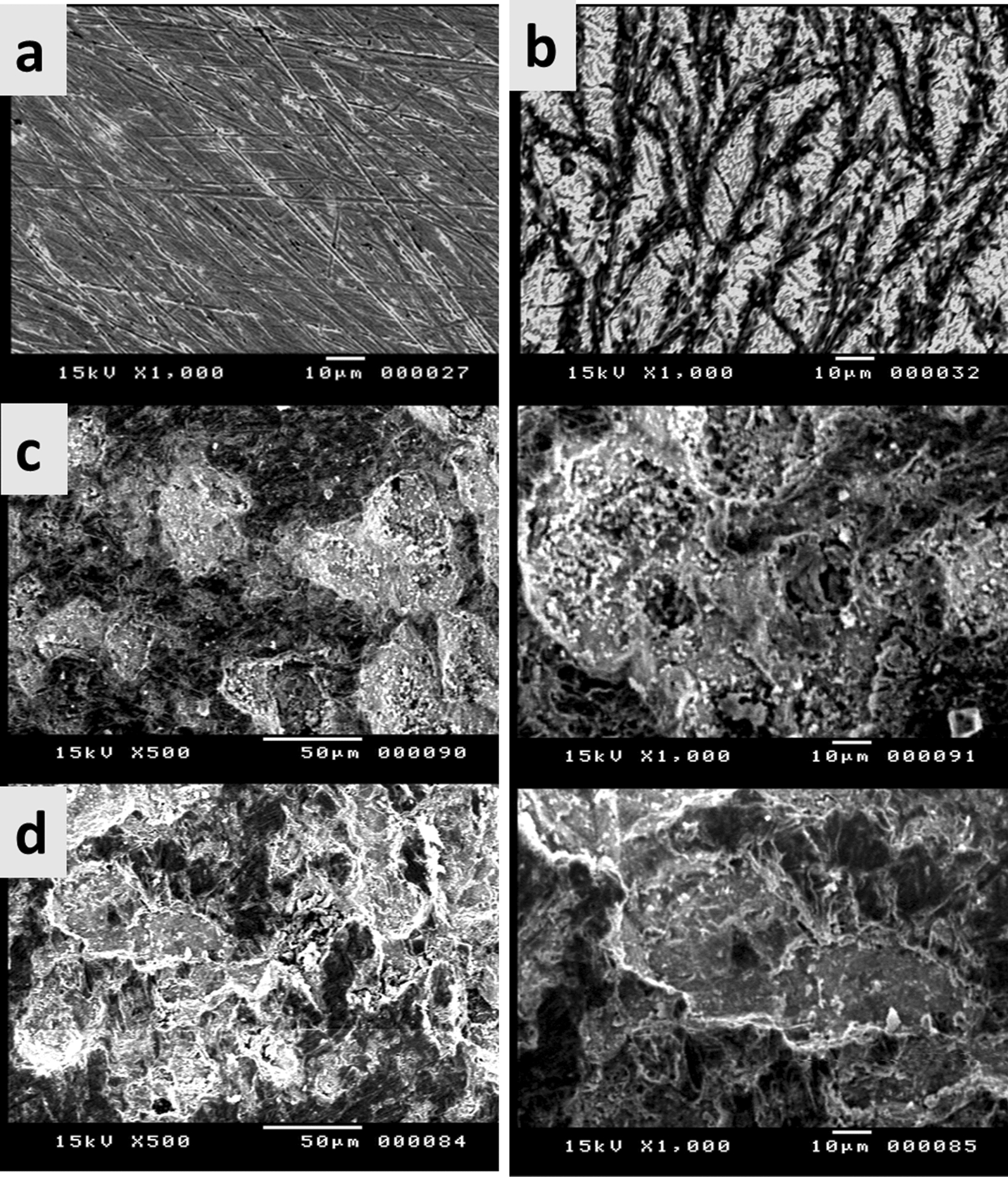
SEM images of mild steel in 1 M H_2_SO_4_ (**a**) without, (**b**) after exposing for 30 min, (**c**) Surface obtained with inhibitor PC6 after exposing for 30 min, and (**d**) Surface obtained with inhibitor PC7 after exposing for 30 min

Correlated impedance profiles can give insight into surface properties and reaction kinetics, making EIS a crucial pre-method in corrosion investigations [61]. (Figure. 6) shows the Nyquist of mild steel in 1.0 M H2SO4 with and without several different polymer samples. As can be seen in (Fig. 6), All of the existing polymeric additives exhibited wider capacitive loops compared to the blank medium, and the protection power exhibited more significant increases with the enhanced, modified polymer, indicating that the polymer layer on the steel interface became denser, leading to the strengthening of MS inhibition. This showed that MS surface inhibition had been enhanced due to increased polymer covering of the metal. Meanwhile, the capacitive loops in all the Nyquist profiles were remarkably similar, proving that the corrosion mechanism was unaffected by the polymer additions. The frequency dispersion also caused incomplete Nyquist profiles, the metal interface's roughness, and heterogeneous nature, adsorbed polymer additives on the MS substrate, and their combined effect [62, 63].

(Figure 7) displays the surface SEM pictures of mild steel before and after being submerged in a 1 M H_2_SO_4_ solution without and with (PCs 6,7). (Fig. 7a, b) shows that the extensive dissolution of mild steel in H_2_SO_4_ solution has severely damaged the metal surface, as seen by the numerous breaks and voids that have developed all over the steel. In contrast, the mild steel corroded less when PC was added to the environment (Fig. 7c, d). The mild steel surface has been treated with inhibitors by adsorption., improving the surface's facial structure and offering a remarkable level of corrosion protection by slowing down metal dissolution. The organic layer in the SEM pictures supports the findings of the electrochemical tests covered in the earlier sections [63].

Natural and synthetic polymers are added to β-cyclodextrin (β-CD) corrosion inhibitors to improve their efficacy by forming simple ether bonds between them and externally active hydroxyl functional groups. Based on the data in (Table 4), the results demonstrate that forming O-C bonds is responsible for most β-CD modifications. Whether in an acidic or basic environment, the polymers can impede the activity of various metals, making them an intriguing guest for the β-CD host. More stability is achieved in host–guest (supramolecular) systems based on polymer β-CDs because of the contacts, H bonds, and noncovalent interactions between the host and guest. (Table 4) shows PC5-7 and other inhibitors' corrosion inhibitory capacity (CD). As was demonstrated, the achieved inhibition efficiency in this study was much higher than that seen in the literature [64–70]. This result demonstrates the PCs' promising capability of inhibiting mild steel.

**Table 4:** Main characteristics of the β-CD modified with synthetic polymers as corrosion inhibitors

Name of inhibitor	Metal type	IE%	Ref.
poly(AM-co-A-β-CD-co-AE) polymer	Carbon steel	90	65
β-cyclodextrin polyethylene glycol (β-CDePEG)	Q235 Carbon steel	97	66
β-cyclodextrin grafted polyacrylamide	N80 Carbon steel	91.3	67
Soluble cyclodextrin polymer (SCDP)	Mild steel	92.2	68
β-cyclodextrin–polyethylene glycol (β-CD–PEG)	Steel	89.1	69
Weak crosslinking cyclodextrin polymer and trans-cinnamaldehyde	Mild steel	92.2	70
β-Cyclodextrin-modified acrylamide polymer (poly(AM-co-A-b-CD-co-NaAA))	X70 steel	84.9	71
Arylidene copolymer with (β-CD)	Mild steel	99.9	This work

## Conclusion

This study is about making and using green, long-lasting anti-corrosion agents derived from β-CD compounds to protect mild steel from corrosion. The research and development of β-CD-based compounds as corrosion inhibitors are crucial for making the next generation of corrosion-resistant materials. This paper describes a simple and effective approach for manufacturing novel pseudopolyrotaxane copolymers (PC5-7) by threading β-CD through the chain (CoP5-7). Tafel polarization and electrochemical impedance spectroscopy (EIS) have been used to estimate anti-corrosion effectiveness IE%; PC7 has the highest inhibitor, at 99.93% in 1.0 M H_2_SO_4_; these inhibitors are mixed-type. FT-IR spectroscopy analysis was employed to establish the chemical structures of the produced polymers. The morphology of the CoPs and PCs was studied using SEM. Compared to the prepared (CoP5-7), the SEM images demonstrate distinct morphological shapes for the prepared (PC5-7). According to X-RD, the addition of complex formation between β-CD and the (CoP5-7) increased the crystallinity of the resultant PCs. TGA studies of their thermal stabilities reveal the production of PCs with higher and modified thermal stability in PC7 due to the increased connection between β-CD and CoP7. The corrosion inhibition of PC5-7 is investigated and found to be suitable for usage as mixed-type inhibitors, with increased mild steel inhibition reaching its highest value in PC7.

## Data Availability

All data generated or analyzed during this study are included in this published article.
